# ZIP8 Is Upregulated in the Testis of *Zip14*^-/-^ Mice

**DOI:** 10.3390/nu16213575

**Published:** 2024-10-22

**Authors:** Varalakshmi Vungutur, Shannon M. McCabe, Ningning Zhao

**Affiliations:** School of Nutritional Sciences and Wellness, The University of Arizona, Tucson, AZ 85721, USA

**Keywords:** manganese, testis, male reproduction, ZIP8, ZIP14, ZnT10

## Abstract

Background/Objectives: Manganese is an essential nutrient involved in various biological processes, including reproductive health, yet the mechanisms regulating its homeostasis in the testis remain poorly understood. Methods and Results: In this study, we investigated the expression and regulation of key manganese transporters—ZIP8, ZIP14, and ZnT10—in mouse testes. Immunoblotting analyses revealed that ZIP8 is expressed in the testes, while ZIP14 and ZnT10 were undetectable. Using *Zip14* knockout (*Zip14*^-/-^) mice, which exhibit systemic manganese overload, we discovered a significant increase in manganese levels in the testis, accompanied by an upregulation of ZIP8. Importantly, the levels of other essential metals, such as iron, zinc, and copper, remained unchanged. Conclusions: Our findings suggest that ZIP8 plays a critical role in manganese transport in the testis, and its increased expression may contribute to manganese accumulation in the absence of ZIP14. This study advances our understanding of manganese homeostasis in the testis and its potential impact on male reproductive health.

## 1. Introduction

Manganese is an essential trace metal that functions as a cofactor for numerous enzymes involved in energy metabolism, antioxidant defense, and immune response [1,2]. For example, arginase, a manganese-dependent enzyme, catalyzes the conversion of L-arginine to L-ornithine and urea, the final step of the urea cycle, which is essential for preventing excess ammonia buildup [3,4]. Glycosyltransferases, which also depend on manganese, are involved in the synthesis of proteoglycans, necessary for cartilage and bone formation [5]. Prolidase is a manganese-activated enzyme that recycles the amino acid proline for collagen synthesis and cell growth [6]. Manganese superoxide dismutase (MnSOD), found in the mitochondria, facilitates the conversion of superoxide radicals into hydrogen peroxide, serving as a crucial antioxidant defense mechanism [7].

Manganese is abundant in various foods, such as legumes, leafy vegetables, fruits, and whole grains. However, manganese deficiency can still occur in individuals with severe malnutrition or certain genetic predispositions, resulting in impaired growth, osteoporosis, and cognitive defects [8]. Conversely, manganese toxicity, known as manganism, typically arises from occupational over-exposure in industries such as mining, welding, and smelting, leading to neurological and behavioral abnormalities; in addition, certain genetic defects can also lead to manganese overload even with normal exposure levels [9]. Therefore, maintaining manganese homeostasis is crucial for overall health. Systemic manganese homeostasis is regulated primarily by the intestine and liver [9,10,11]. Once dietary manganese is absorbed in the intestine, it enters the bloodstream and is transported to the liver via the portal vein. In the liver, manganese can either be stored, transported to other tissues, or secreted into bile for excretion. Any unabsorbed manganese in the intestine is eventually eliminated through fecal excretion [1,12]. This coordinated effort ensures proper manganese levels are maintained in the body, balancing intake and excretion to prevent deficiency or toxicity.

Certain organs, including the male reproductive system, have a particularly high demand for manganese to support their specialized functions. Manganese is required for the synthesis of steroid hormones, the regulation of oxidative stress, and the maintenance of normal spermatogenesis [10,13,14]. Adequate manganese levels are crucial for testicular function, including the development and maturation of sperm cells, and for protecting reproductive tissues from oxidative damage [15,16]. Formaldehyde (FA) exposure in mice significantly reduced testicular weight, sperm count, motility, viability, and normal morphology while causing seminiferous tubule atrophy and epithelial cell disintegration. Manganese, administered intraperitoneally at 5 mg/kg during the second week, improved sperm parameters and testis structure in FA-exposed mice, likely by mitigating FA-induced oxidative stress [15]. Another study examined the protective role of manganese during cryopreservation of cattle bull semen by assessing its impact on oxidative stress and sperm quality. Manganese, added to an egg-yolk-citrate extender with glycerol at concentrations of 100, 150, and 200 µM, significantly improved sperm motility, hypo-osmotic swelling, and reduced lipid peroxidation (LPO) and protein leakage, particularly at 150 and 200 µM after freezing. These findings suggest that manganese acts as an antioxidant, reducing oxidative stress and enhancing semen quality, which could improve fertility outcomes in artificial insemination and in vitro fertilization [16]. Therefore, maintaining balanced manganese levels is vital not only for overall health but also for male fertility and reproductive health. However, while adequate levels of manganese are essential for male reproductive health, excessive manganese can have detrimental effects [17]. Excess manganese has been shown to disrupt initial testicular development by damaging the integrity of Sertoli cells through oxidative stress [18]. Since the Sertoli cells are critical for supporting and nurturing developing sperm cells, this oxidative insult may compromise spermatogenesis over the long term, potentially leading to impaired fertility and other reproductive issues [17,19]. 

Despite the importance of manganese in maintaining the proper function of male reproduction, the mechanisms regulating manganese homeostasis in the testis remain poorly understood. Central to this regulation are manganese transporters, which facilitate the uptake, distribution, and excretion of manganese at the cellular level. Several manganese transporters have been identified, including ZIP8, ZIP14, and ZnT10 [9]. ZIP8 and ZIP14 are members of the Zrt- and Irt-like protein (ZIP) and are responsible for bringing manganese into cells [20,21,22], while ZnT10 belongs to the zinc transporter (ZnT) family and helps remove manganese from cells [23,24]. However, the specific expression patterns and roles of these transporters in the testis have not been thoroughly investigated. Determining which transporters are active in the testis and their functions is fundamental to elucidating the mechanisms by which manganese affects testicular development, spermatogenesis, and overall male reproductive health.

This study aims to identify and characterize the expression of manganese transporters in the testis to elucidate their potential roles in maintaining manganese homeostasis in the testes. By shedding light on these mechanisms, we hope to provide new insights into the role of manganese in reproductive health and identify potential targets for addressing manganese-related reproductive disorders.

## 2. Materials and Methods

**Animals and tissue collection**

All animal experiment procedures were approved by the Institutional Animal Care and Use Committees at the University of Arizona (approval code: 16-172; approval date: 25 July 2022). Mice were housed in cages with a maximum of five mice per cage, maintained at a temperature of 21–22 °C, and subjected to a 12-h light/dark cycle. The mice had ad libitum access to tap water. At three weeks of age, the mice were weaned and placed on a standard rodent diet (Envigo, Indianapolis, IN, USA). To generate inducible *Zip8* knockout (*Zip*8-iKO) mice, animals carrying the *Zip8* conditional allele (*Zip8*^flox/flox^) (purchased from Taconic, Germantown, NY, USA) were crossbred with mice expressing the Cre-ERT2 (tamoxifen-inducible estrogen receptor fusion protein) under the control of the ubiquitin promoter (*Ubc*-Cre) (obtained from the Jackson Laboratory, Bar Harbor, ME, USA). Both *Zip8*^flox/flox^ and *Ubc*-Cre mice were from the C57BL/6 background. *Zip8*^flox/flox^-Ubc-Cre mice were injected with tamoxifen dissolved in corn oil (100 mg/kg body weight) for 5 consecutive days to inactivate *Zip8*. The *Zip14* knockout mice are on the 129/Sv background. We used heterozygous mice as the breeding pairs, ensuring that the wild-type mice used in the experiments were also of the same background. All mice were sacrificed following anesthesia with ketamine/xylazine. Mouse tissues were immediately frozen in liquid nitrogen and stored at −80 °C for further analyses.

**Metal level determination**

Metal contents in mouse testis tissues were analyzed using inductively coupled plasma mass spectrometry (ICP-MS) at the Arizona Laboratory for Emerging Contaminants (ALEC). Frozen testes were dried over 3 days at 80 °C until the tissue weights were stabilized. The dried tissues were then digested with 2 mL of 70% concentrated HNO_3_ overnight at room temperature and then at 80 °C for 6 h. Digested tissue samples were then cooled down at room temperature. A quantity of 400 μL of the tissue digest was diluted with 9 mL of Milli-Q water with the final total volume being 9.4 mL and the final concentration of HNO_3_ being 3%. Samples were then sent to the ALEC lab and metal concentrations were calculated to find the μg of metal/μg of dry tissue. 

**Immunoblotting**

Mouse testis tissues were homogenized in ice-cold NETT lysis buffer (150 mM NaCl + 5 mM EDTA + 10 mM Tris + 1% Triton X-100 in deionized water) containing 1× Protease inhibitor (Bimake, Houston, TX, USA). The homogenized samples were then centrifuged at 10,000× *g* for 10 min at 4 °C, and the supernatant was collected to be used in the immunoblotting Analysis. The protein concentration of the lysed samples was determined using the Reducing Agent and Detergent Compatible (*RC DC*) protein assay (Bio-Rad Life Science, Hercules, CA, USA), following the manufacturer’s protocol. Samples with equal amounts of protein were mixed with 1× Laemmli buffer, and then incubated at 37 °C for 30 min. Proteins were electrophoretically separated on 10% sodium dodecyl sulfate polyacrylamide gel and transferred to nitrocellulose membrane (GVS, Sanford, ME, USA). Membranes were blocked with blocking buffer [5% non-fat dried milk in TBST (tris-buffered saline + 0.1% Tween 20)] for 1–3 h at room temperature and incubated with rabbit anti-mouse ZIP8, ZIP14 or ZnT10 antibodies (1:1000) at 4 °C overnight (these primary antibodies were generated by our laboratory using the same process as our published protocol for the ZIP8 antibody generation [25]. Following four washes with TBST (5 min per wash), membranes were incubated with horseradish peroxidase (HRP)-conjugated anti-rabbit secondary antibody (1:3000, GE Healthcare, Chicago, IL, USA). Prior to imaging, membranes were washed twice with TBST and then twice with TBS (5 min per wash). Signal was detected using an enhanced chemiluminescent substrate (SuperSignal West Pico, Thermo Fisher Scientific, Waltham, MA, USA) and ChemiDoc MP Imaging System (Bio-Rad Life Science, Hercules, CA, USA). The blots were quantified using the Image Lab 6.1 software (Bio-Rad Life Science, Hercules, CA, USA). For the gel loading control, membranes were re-probed with an HRP-conjugated anti-β-Actin antibody (1:6000, Proteintech, Rosemont, IL, USA). 

**Statistical Analysis**

Data analysis was conducted using GraphPad Prism 8.0 (GraphPad Software, San Diego, CA, USA). The F-test was utilized to assess equal variances between groups. Comparisons of ZIP8 levels between groups were performed using an unpaired Student’s *t*-test. Statistical significance was determined with a *p*-value of less than 0.05.

## 3. Results

### 3.1. Validation of Antibodies Used in the Western Blotting Analysis

**Figure 1:**
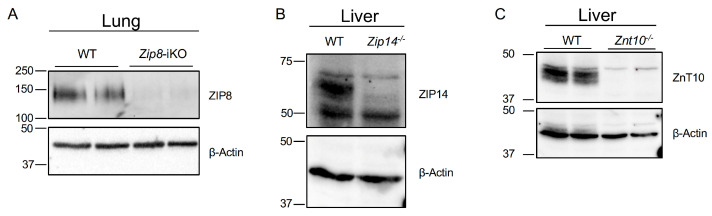
Validation of antibodies used in immunoblotting analyses. (**A**) Immunoblotting analysis of ZIP8 expression in lung tissues from wild-type (WT) and tamoxifen-induced *Zip8* knockout (*Zip8*-iKO) mice. The anti-ZIP8 primary antibody shows specific detection of ZIP8 protein in WT lungs, with no signal observed in *Zip8*-iKO lungs. (**B**) Immunoblotting analysis of ZIP14 expression in liver tissues from WT and *Zip14* knockout (*Zip14*^-/-^) mice. (**C**) Immunoblotting analysis of ZnT10 expression in liver tissues from WT and *Znt10* knockout (*Znt10*^-/-^) mice. These results confirm the specificity of the antibodies used for ZIP8, ZIP14, and ZnT10 in immunoblotting.

We aim to identify the protein expression of ZIP8, ZIP14, and ZnT10 in the testes using immunoblotting analysis. Since antibody specificity is critical in immunoblotting, we first used gene knockout mouse tissues as negative controls to ensure accurate detection of the target proteins. The global knockout of *Zip8* in mice is embryonically lethal, therefore we used the *Zip8*^flox/flox^-Ubc-*Cre* mice to generate a tamoxifen-inducible *Zip8* knockout (*Zip8*-iKO). Previous studies have demonstrated that ZIP8 protein is highly expressed in the lungs [25,26]. Therefore, we used lung samples from both wild-type and *Zip8*-iKO mice to confirm the efficacy of the anti-ZIP8 primary antibody (Figure 1A and Figure S1). The liver expresses high levels of ZIP14 [27,28] and ZnT10 [29,30], liver samples were used to confirm the efficacy of the antibodies for these two proteins, with *Zip14* knockout (*Zip14*^-/-^) and *Znt10* knockout (*Znt10*^-/-^) samples serving as negative controls, respectively (Figure 1B,C and Figure S1). We confirmed the specificity of antibodies for ZIP8, ZIP14, and ZnT10, with no signal detected in the corresponding knockout samples. 

### 3.2. Expression of Manganese Transporters in Mouse Testes

**Figure 2:**
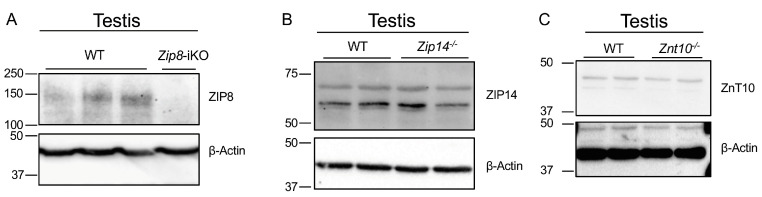
Expression of manganese transporters in the testis. (**A**) Immunoblotting analysis of ZIP8 expression in testes from WT and *Zip8*-iKO mice. A distinct ZIP8 band is observed in WT testis but is absent in *Zip8*-iKO testis, confirming the expression of ZIP8 in the testis. (**B**) Immunoblotting analysis of ZIP14 expression in WT and *Zip14*^-/-^ testes. No detectable signal for ZIP14 is observed, suggesting minimal or no expression of ZIP14 in the testis. (**C**) Immunoblotting analysis of ZnT10 expression in WT and *Znt10*^-/-^ testes. Similar to ZIP14, no detectable ZnT10 signal is observed, indicating that ZnT10 is either not expressed or is below the detection limit in testicular tissue.

To better understand the role of manganese in testicular function, fertility, and overall reproductive health, we investigated which of the three manganese transporters—ZIP8, ZIP14, and ZnT10—are expressed in mouse testes. Immunoblotting analyses revealed that ZIP8 is expressed in the testis of wild-type mice, evidenced by a distinct ZIP8 band that was absent in *Zip8*-iKO mice (Figure 2A and Figure S2). In contrast, no differences in band patterns were observed for ZIP14 and ZnT10 between wild-type and their respective knockout samples (Figure 2B,C and Figure S2). The lack of differences in band patterns for ZIP14 and ZnT10 between wild-type and knockout samples suggests that these proteins are barely detectable because, in knockout models, we would expect a significant reduction or absence of protein expression. The presence of faint bands in both wild-type and knockout samples likely indicates that the protein levels are too low to produce discernible changes in band intensity, leading to the conclusion that these proteins are present at very low levels or are not sufficiently detected by this method.

After identifying ZIP8 expression in the testis, we then aimed to determine how its regulation is affected by high manganese levels. Since ZIP8 is a key manganese transporter, understanding how its expression responds to elevated manganese concentrations will help clarify its role in maintaining manganese homeostasis within the testes, which could impact testicular function, oxidative stress management, and overall male reproductive health.

### 3.3. Testis Manganese Increases in Zip14 Knockout Mice

To study the regulation of ZIP8 in the testis under conditions of high manganese, we sought to use a genetic manganese overload mouse model, *Zip14*^-/-^ mice. These mice exhibit significantly increased manganese levels in the blood and brain, along with reduced manganese levels in the liver [31,32,33]. However, the manganese content in the testes of *Zip14*^-/-^ mice has not yet been determined, representing a critical knowledge gap. Therefore, we first investigated whether these mice show changes in manganese content within the testes.

**Figure 3:**
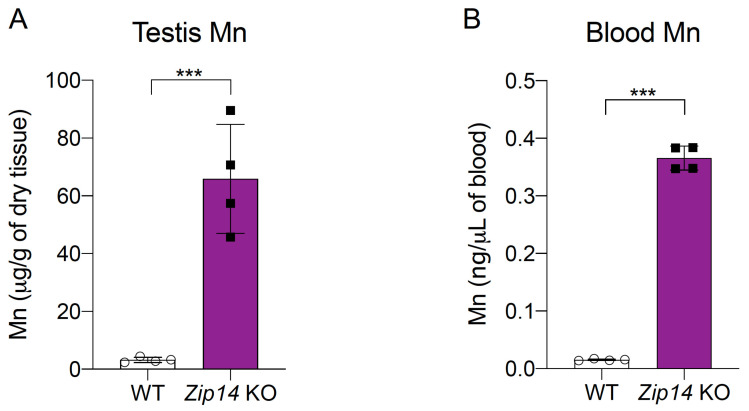
Testis manganese levels in *Zip14*^-/-^ mice. (**A**) Inductively coupled plasma mass spectrometry (ICP-MS) analysis of manganese content in the testes of WT and *Zip14*^-/-^ mice. (**B**) ICP-MS analysis of blood manganese levels in WT and *Zip14*^-/-^ mice. Data are expressed as mean ± standard deviation (SD) (*n* = 4). *** *p* < 0.001.

ICP-MS analysis revealed that *Zip14*^-/-^ mice have over 20 times higher manganese levels in their testes compared to control animals (3.229 ± 0.916 μg/g vs. 65.85 ± 18.82 μg/g) (Figure 3A). Consistent with previous studies [31,32,33], blood manganese levels in *Zip14*^-/-^ mice showed a similar extent of manganese loading (0.0153 ± 0.0013 μg/mL vs. 0.3656 ± 0.0209 μg/mL) (Figure 3B). This finding is significant because it reveals, for the first time, a substantial increase in manganese levels in the testes of *Zip14*^-/-^ mice, suggesting that ZIP14 may be indispensable for regulating manganese transport into the testes and maintaining testicular manganese homeostasis. Importantly, this result also confirms that *Zip14*^-/-^ mouse model is suitable for studying ZIP8 regulation in the testes under high manganese conditions.

### 3.4. Levels of Iron, Zinc, or Copper in Zip14 Knockout Mice Do Not Differ from That of the Wild-Type Animals

**Figure 4:**
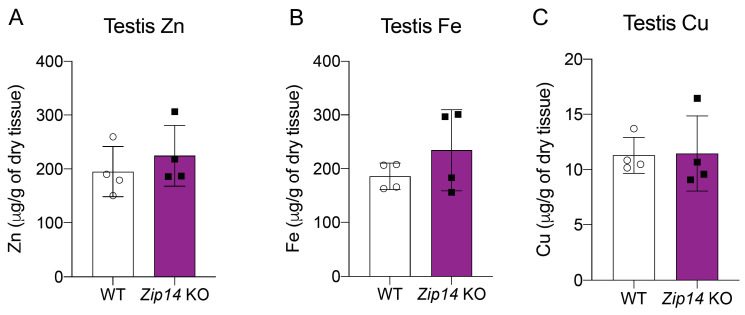
Levels of zinc, iron, and copper in the testes of *Zip14*^-/-^ mice. (**A**) Zinc, (**B**) Iron, and (**C**) copper levels in the testes of WT and *Zip14*^-/-^ mice, measured by ICP-MS. No significant difference in the levels of these three metals is observed between WT and *Zip14*^-/-^ mice. Data are expressed as mean ± SD (*n* = 4).

ZIP14 is known to transport multiple divalent metals, including zinc and iron [34,35]. After detecting increased manganese levels in *Zip14*^-/-^ mice, we investigated whether levels of other essential metals were also affected. Our analysis revealed no significant differences in zinc and iron levels in the testes between wild-type and *Zip14*^-/-^ mice (Figure 4A,B), suggesting that the loss of ZIP14 does not disrupt the homeostasis of these metals in testicular tissues. Given a previous study showing that manganese exposure significantly alters systemic copper levels [33], we also measured copper concentrations in the testes of *Zip14*^-/-^ mice. We found that copper levels remained unchanged (Figure 4C). Together, these results demonstrate that the increase in manganese levels observed in *Zip14*^-/-^ mice is specific to manganese transport and not due to alterations in the transport or regulation of other essential metals like zinc, iron, or copper. This ensures that any effects observed on ZIP8 are specifically related to changes in manganese rather than other metal ions. 

### 3.5. ZIP8 Protein Expression Increases in the Testes of Zip14 Knockout Mice

**Figure 5:**
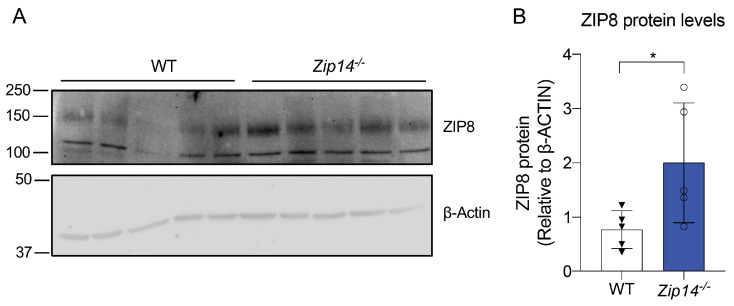
ZIP8 protein expression in the testes of *Zip14*^-/-^ mice increases. (**A**) Immunoblotting analysis of ZIP8 protein levels in testes from WT and *Zip14*^-/-^ mice. (**B**) Quantification of ZIP8 protein expression, normalized to β-Actin, reveals a significant increase in ZIP8 levels in *Zip14*^-/-^ mice. Data are expressed as mean ± SD (*n* = 5). * *p* < 0.05.

To study how high manganese conditions regulate manganese transporters is crucial for understanding manganese homeostasis and preventing toxicity. To investigate how ZIP8 in the testis is regulated by high manganese levels, we performed immunoblotting analysis to measure ZIP8 protein levels. Our results showed that ZIP8 protein levels were significantly increased in *Zip14*^-/-^ mice (Figure 5A,B and Figure S3). This indicates that, in the absence of ZIP14, upregulation of ZIP8 could contribute to the enhanced manganese accumulation observed in the testes. Moreover, the upregulation of ZIP8 in response to ZIP14 loss could point to ZIP8 as a potential therapeutic target in conditions where manganese overload occurs due to dysregulated transporter activity, particularly in reproductive health contexts. 

## 4. Discussion

The testis requires manganese for the synthesis of steroid hormones, the regulation of oxidative stress, and the maintenance of normal spermatogenesis. These roles highlight the critical function of manganese in male fertility and overall reproductive health. In this study, we explored the expression and regulation of manganese transporters in mouse testes to understand their roles in maintaining manganese homeostasis and potential implications for male reproductive health. Using validated antibodies, we identified that ZIP8 is expressed in the testis, while ZIP14 or ZnT10 are not detectable by immunoblotting in this tissue. The use of validated antibodies ensured the specificity and accuracy of our findings, confirming that ZIP8 may play an important role in manganese transport within the testis. One limitation of our study is the reliance on immunoblotting for protein detection. While this method is widely used, it may not be sensitive enough to detect certain proteins, leading to a lack of signal. This can create ambiguity in interpreting whether the absence of a signal reflects true absence of the protein or simply a detection issue. As a result, we have cautiously interpreted our findings, using the term “not detectable” rather than “not expressed” when discussing protein levels in the testis. To overcome this limitation, future studies could incorporate other techniques, such as immunohistochemistry, which would allow for visualization of protein expression and localization within the tissue. 

After identifying the expression of ZIP8 in the testis, we aimed to determine how its regulation is influenced by manganese levels. Understanding its regulation can provide insights into how manganese levels are controlled in the testis, potentially revealing new therapeutic targets for conditions linked to disrupted manganese transport, such as infertility or certain diseases. Additionally, investigating ZIP8’s regulation may uncover unique, tissue-specific mechanisms, highlight its involvement in testicular diseases, and offer broader implications for male reproductive health. 

To investigate how testis ZIP8 is regulated by high manganese, we used *Zip14*^-/-^ mouse model. *Zip14*^-/-^ mice show a varied pattern of tissue manganese levels, with dramatic increases in the brain and bone but decreases in the liver. Due to these varied changes, it is crucial to determine manganese levels in the testis to understand manganese distribution in these mice. We found that manganese levels in the testes of *Zip14*^-/-^ mice are dramatically elevated, addressing a significant knowledge gap in our understanding of manganese distribution in these mice. This finding also suggests that the *Zip14*^-/-^ mouse model is suitable for examining the regulation of ZIP8 in the testis under conditions of high manganese. We further discovered that ZIP8 expression is increased in the testes of *Zip14*^-/-^ mice. The increased expression of ZIP8 in *Zip14*^-/-^ mice indicates that under high systemic manganese conditions, ZIP8 facilitates manganese transport into the testis, which contributes to the accumulation of manganese in this tissue. Importantly, our findings also show that the manganese accumulation in the testes of *Zip14*^-/-^ mice is not accompanied by changes in the levels of other essential metals, such as iron, zinc, or copper, suggesting that the regulation of these metals in the testes is not dependent on ZIP14. This specificity reinforces the idea that any observed changes in ZIP8 are directly related to its role in manganese transport, rather than secondary effects caused by broader disruptions in metal homeostasis. 

## 5. Conclusions

Our study highlights the significant role of ZIP8 in regulating manganese homeostasis in the testis, particularly under conditions of elevated manganese. Given that manganese is both essential and potentially toxic at high concentrations, the regulation of ZIP8 may be a key determinant in preventing metal-induced reproductive dysfunction. Our findings have several implications. The increase in ZIP8 expression may reflect an adaptive response to maintain manganese homeostasis in the testis under conditions of elevated manganese. This could impact testicular function, including spermatogenesis and hormone production, given that manganese is essential but can be toxic at high concentrations. Additionally, these results provide insight into tissue-specific regulation of manganese transport, where ZIP8 appears to be the primary transporter managing manganese uptake in the testis. If similar mechanisms exist in humans, these findings could improve our understanding of reproductive disorders related to metal dysregulation and guide potential therapeutic approaches for managing manganese-related conditions. Further studies should focus on understanding the molecular mechanisms that regulate ZIP8 expression and its interactions with other metal transporters in the testis, which could provide new insights into the management of manganese toxicity and related reproductive disorders.

## Data Availability

The original contributions presented in the study are included in the article/supplementary material, further inquiries can be directed to the corresponding author.

## Supplementary Materials

The following supporting information can be downloaded at: https://www.mdpi.com/article/10.3390/nu16213575/s1, Figure S1. Uncropped immunoblot images shown in Figure 1. Figure S2. Uncropped immunoblot images shown in Figure 2. Figure S3. Uncropped immunoblot images shown in Figure 5A.
