# Supplementary material for: ZIP8 Is Upregulated in the Testis of Zip14-/- Mice
Source: Nutrients. 2024 Oct 22;16(21):3575. doi: 10.3390/nu16213575 (PMC11547875; doi:10.3390/nu16213575)

nutrients

Article

ZIP8 is upregulated in the testis of *Zip14*$^{-/-}$ mice

Varalakshmi Vungutur, Shannon M. McCabe, and Ningning Zhao*

* Correspondence: zhaonn@arizona.edu; Tel.: (520) 621-9744

Supplemental Figures S1, S2 and S3
(Uncropped Immunoblot Images for Figure 1, 2 and 5A)

Figure S1. Uncropped immunoblot images shown in Figure 1.

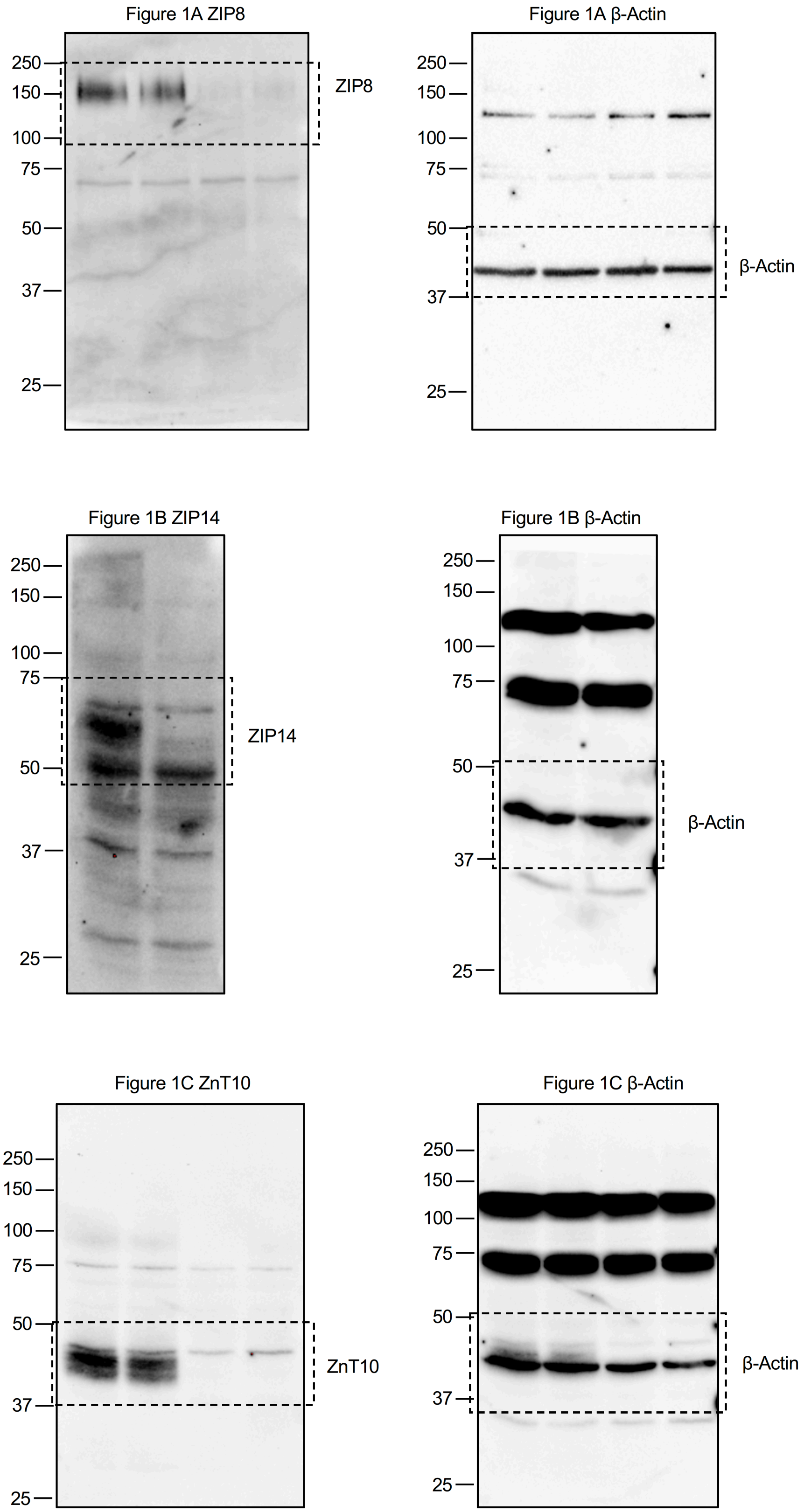

Figure S2. Uncropped immunoblot images shown in Figure 2.

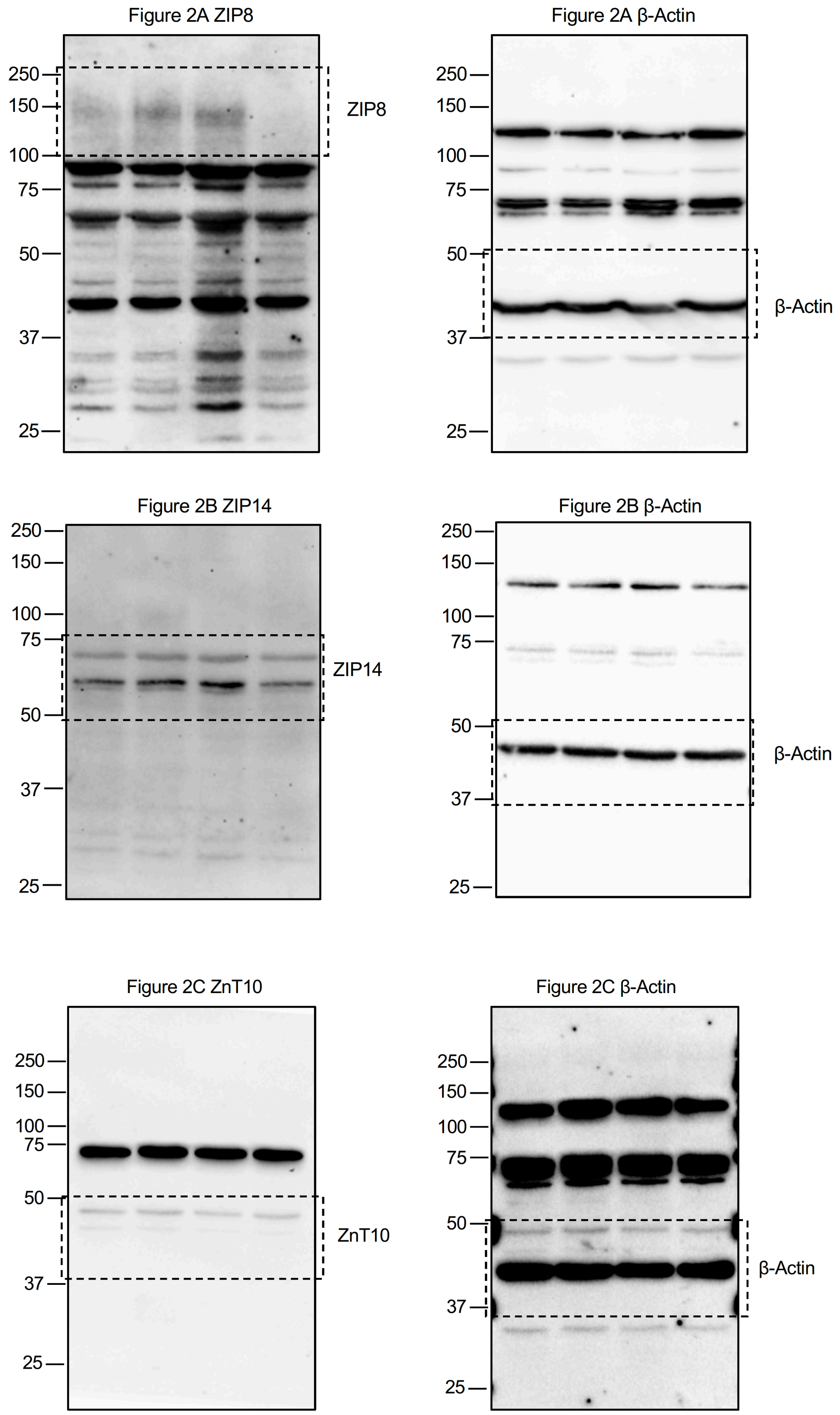

Figure S3. Uncropped immunoblot images shown in Figure 5A.

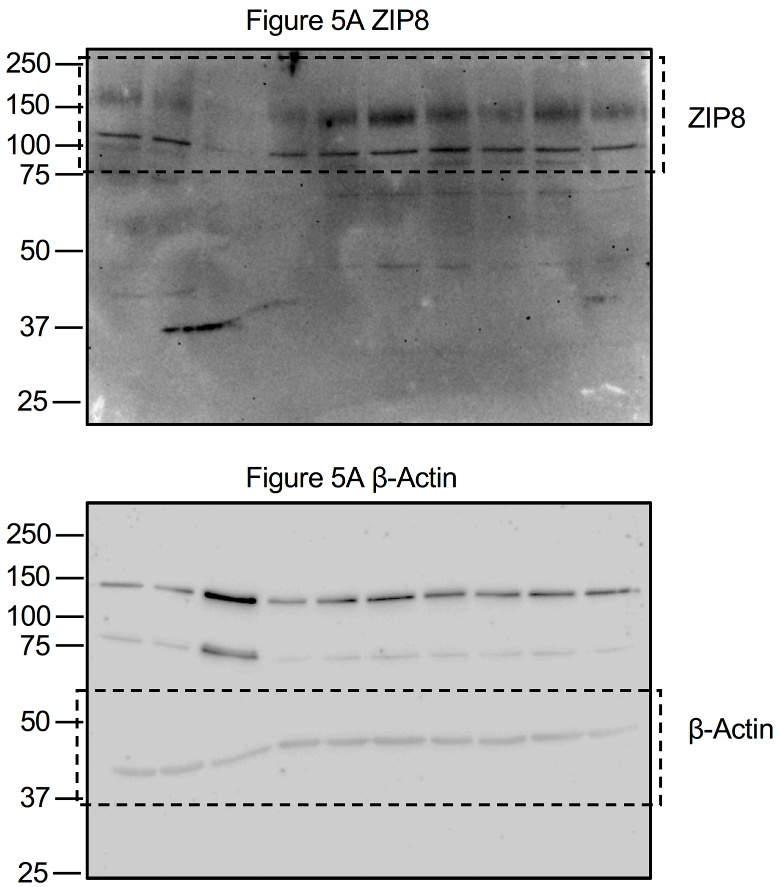

Supplement: Supplementary file 1 [file nutrients-16-03575-s001.zip › nutrients-3245152-supplementary.pdf]
